# Neonatologist Performed Echocardiography for Evaluating the Newborn Infant

**DOI:** 10.3389/fped.2022.853205

**Published:** 2022-03-24

**Authors:** Eirik Nestaas

**Affiliations:** ^1^Institute of Clinical Medicine, University of Oslo, Oslo, Norway; ^2^Clinic of Pediatrics and Adolesence, Akershus University Hospital, Nordbyhagen, Norway

**Keywords:** heart function, neonatologist performed echocardiography, neonatal intensive care, point-of-care ultrasound (POCUS), clinician-performed ultrasound, focused cardiac ultrasound (FocUS), targeted neonatal echocardiography

## Abstract

The interest in the use of cardiac ultrasound for hemodynamic evaluation in neonates has increased in the last decades. Several overlapping terms exists, and a non-comprehensive list includes point-of-care ultrasound, clinician-performed ultrasound, focused cardiac ultrasound, targeted neonatal echocardiography, and neonatologist performed echocardiography. This review will use the term neonatologist performed echocardiography. Neonatologist performed echocardiography is primarily echocardiography to obtain snapshots of hemodynamics and heart function, usually as repeated exams during intensive care. It provides the neonatologist with in-depth information on the hemodynamics not available by blood pressure, pulse oximetry, capillary refill time, and various blood tests. The review provides a brief overview of some relevant methods for assessment of hemodynamics and heart function. It does not discuss training, implementation, accreditation, and certification nor in-depth technical aspects and detailed use of neonatologist performed echocardiography. If the information obtainable by neonatologist performed echocardiography had been accessible easily (e.g., via a sensor put on the neonate similarly to a pulse oximeter), neonatologist performed echocardiography would have been more widely used. Acquiring skills for neonatologist performed echocardiography take time and resources. Future developments probably include a stronger focus on education, training, and certification. It is likely that echocardiographic methods will evolve further, probably involving establishing new indexes and methods and implementing artificial intelligence in the analyses procedure to improve accuracy and workflow. It is important to acknowledge that neonatologist performed echocardiography is not a therapeutic intervention; it is a diagnostic tool providing additional information.

## Introduction

The interest in use of cardiac ultrasound for hemodynamic evaluation in neonates has increased the last decades. Several overlapping terms exists. A non-comprehensive list includes point-of-care ultrasound, clinician-performed ultrasound, focused cardiac ultrasound, targeted neonatal echocardiography, and neonatologist performed echocardiography (NPE). This review will use the term NPE, in line with a recent series of publications on the topic (1–9). NPE is primarily echocardiography to obtain snapshots of hemodynamics and heart function, usually as repeated exams during intensive care. It provides the neonatologist with in-depth information on the hemodynamics not available by blood pressure, pulse oximetry, capillary refill time, and various blood tests.

This article provides a brief overview of some methods relevant for NPE. It does not discuss training, implementation, accreditation, and certification nor in-depth technical aspects and detailed of use of NPE. Several papers (1–8, 10–14) and websites (15–17) provide reference values and more information. The supplement file shows examples of some of the established and experimental methods used in NPE.

## Elements of a NPE Exam

A NPE exam will usually consists of several elements, frequently evaluating systemic blood flow, pulmonary vascular resistance/hypertension, ventricular function, and presence of pericardial effusion and fetal shunts. In a newborn with compromised circulation, it is paramount to rule out congenital heart diseases. If congenital heart disease is present, further diagnostics and treatment should be a close teamwork between pediatric cardiologists and neonatologists.

### Assessment of Intravasal Fluid Volume and Systemic Blood Flow

#### Technical Aspects

Velocity–time curves and cross-sectional areas form the basis for calculating blood flow by echocardiography. The most common flow assessments in NPE are at the aortic and pulmonary valve orifices, with aortic velocity–time curves assessed by pulsed Doppler from apical five-chamber or apical long axis, and pulmonary curves from parasternal short axis. Prerequisites for accurate measurements include (1) assessing velocities and cross-sectional areas at the same position, (2) avoiding angle errors (including out-of-plane errors), and (3) avoiding measurements in close succession to relative stenosis, i.e., areas with blood flow unevenly distributed within the vessel. Averaging measurements over several heartbeats improves reproducibility. Variability in cross-section areas is probably the most important cause for the relatively poor reproducibility of 30% (18, 19). The calculations usually use linear dimension (diameters) for estimating cross-sectional areas, and errors in the linear dimensions will lead to squared errors in flow estimates.

Vena cava superior drains blood from the upper part of the body, and a large proportion is cerebral blood flow. Flow in this vessel may hence serve as a surrogate for cerebral blood flow and has the advantage that the fetal shunts will not introduce bias in the estimates. The most common method assesses the cross-sectional area of the vein by use of the diameter of the vessel from the parasternal intermediate axis and the velocity–time curve from the subcostal view (20). Prerequisites for flow estimation by echocardiography are probably met to a less extent in venous than arterial vessels. The calculation of blood flow assumes that blood in the entire cross-sectional area moves at the speed drawn as the outer edge of the velocity–time curves and that the cross-sectional shape of the vessel is circular. As software packages allows for assessing cross-sectional areas directly on images, it is possible to measure the actual cross-sectional area as an alternative to calculating it from linear dimensions (21, 22).

#### Clinical Aspects

A frequent clinical issue in neonatal intensive care is evaluation of fluid depletion. There are no established gold standard by echocardiography for assessing intravascular hypovolemia and showing a need for fluid therapy in newborns. However, NPE may follow the effects of therapy, e.g., changes in systemic blood flow after a therapeutic intervention. Blood flow over the aortic and pulmonary valves may serve as measures of systemic blood flow if the fetal shuts are closed. If the fetal shuts are open, the magnitude and direction of the blood flow through the shunts can have significant impact on pulmonary and aortic valve blood flow as indexes of systemic blood flow. However, if the shunts are closed or show left–right flow, systemic blood flow will be acceptable in most premature infants first 2 days of life if the peak flow of the pulmonary valve is >0.45 m/s (23). Normal systemic blood flow in neonates are within the range of 150–300 ml/kg/min and normal blood flow in vena cava superior 40–150 ml/kg/min.

### Blood Pressure and Vascular Resistance in the Pulmonary Circulation

#### Technical Aspects

It is possible to estimate pressure gradients from blood flow velocities by use of the simplified Bernoulli equation, where maximum pressure difference (in mm Hg) is 4 × peak velocity (m/s)^2^. The most common measurement is the peak velocity of the tricuspid valve regurgitation, as an estimate of systolic pressure in the right ventricle and as a proximate of pulmonary artery systolic pressure. The peak diastolic regurgitation of the pulmonary valve may serve as an estimate of pulmonary artery diastolic pressure. The Bernoulli equation is only valid if the narrow part is short, meaning that one should be careful quantifying pressure difference between the pulmonary and systemic circulation based on velocities in the ductus arteriosus. Adding estimates of right atrial pressure and right ventricular diastolic pressure (typically 5 mmHg) probably improves accuracy of measurements as estimates of the right ventricular systolic pressure by the tricuspid valve regurgitation and diastolic pulmonary pressures by the pulmonary valve regurgitation, respectively. Importantly, especially for high velocities, even minor errors in velocity measurements will have high impact on pressure estimates.

Recently, the time between onset and peak pulmonary valve velocity (pulmonary arterial acceleration time, PAAT) as fraction of right ventricular ejection time (RVET) has again gained interests as a non-invasive measure of pulmonary blood pressure, vascular resistance, and afterload (24). Accurate estimates require velocity–time curves of high quality.

#### Clinical Aspects

Evaluation of blood pressure and vascular resistance in the pulmonary circulation is very relevant during intensive care in neonates, as an isolated phenomenon and relative to the left side. The ventricular septum normally bulges into the right ventricle in the short axis view at the papillary muscle level. With increasing pressure equilibrium, the ventricular septum may flatten and even bulge into the left ventricle cavity. Changed shape in diastole is a marker for diastolic pressures and septal shape in systole a marker for systolic pressures. High velocity of the tricuspid regurgitation is a marker for elevated systolic pressure gradient between the right atrium and ventricle. PAAT/RVET decreases with increasing pulmonary vascular resistance. Values larger than 0.31 are considered normal and below 0.23 a marker of high pulmonary vascular resistance (2).

In case of persistent fetal shunts, direction and velocity of blood flow indicate pressure differences between the right and left side. Importantly, ductus arteriosus flow velocity might underestimate the true pressure gradient because of angle errors and because the narrow part of the vessel is too long for meeting the prerequisites of the calculation.

### Ventricular Function

#### Technical Aspects

##### Cavity-Based Indexes

Shortening fraction and ejection fraction of the left ventricle are the most common cavity-based indexes of heart function. Shortening fraction is the percentage change in diameter of the left ventricle from end-diastole to end-systole, obtained from parasternal views at the tip of the mitral valve (25). Ejection fraction is the percentage change in left ventricular volume from end-diastole to end-systole, usually calculated by use of the Simpson biplane method from the apical two- and four-chamber views (25). There are several cavity-based indexes of right ventricle function. The cavity has a complex geometrical shape. Most indexes hence will only assess the function within a region of the ventricle. Many therefore still use qualitative visual assessments (“eye-balling”) from apical and parasternal views. Among available indexes, right ventricle fractional area change is probably most commonly used. It is the percent change in area of the right ventricle from an apical four-chamber view between end-diastole and end-systole (25). However, due to the complex right ventricular cavity shape, the index will not assess function in the outlet part of the cavity (infundibulum).

##### Myocardial Performance Index

There are several time indexes of heart function, but none are widely used in NPE. The Myocardial Performance Index (MPI) is probably the time index most extensively studied. It is the duration of the isovolumic phases of the cardiac cycle, divided by the duration of the ejection phase (26). Its role as an index of heart function in neonates remains unclear.

##### Atrioventricular Plane Motion and Velocities

Apical M-mode can assess the excursion of the atrioventricular plane as indexes of longitudinal function. Tricuspid annular plane systolic excursion (TAPSE) is the excursion of the lateral hinge of the tricuspid valve as an index of right ventricle. Similarly, mitral annular plane systolic excursion (MAPSE) is the excursion on the left side. Some calculate MAPSE as the average of excursion of the septal and lateral hinges of the mitral valve, whereas others measure them as the excursion of the lateral hinge.

Tissue Doppler can assess atrioventricular plane velocities from the apical four-chamber view (7). There are two tissue Doppler modalities, namely, pulsed-wave tissue Doppler and color tissue Doppler. Velocity measurements are not interchangeable between modalities. Only color tissue Doppler can measure displacement of the atrioventricular plane, which is the tissue Doppler equivalence of TAPSE and MAPSE.

##### Deformation and Rotational Indexes

**Figure 1:**
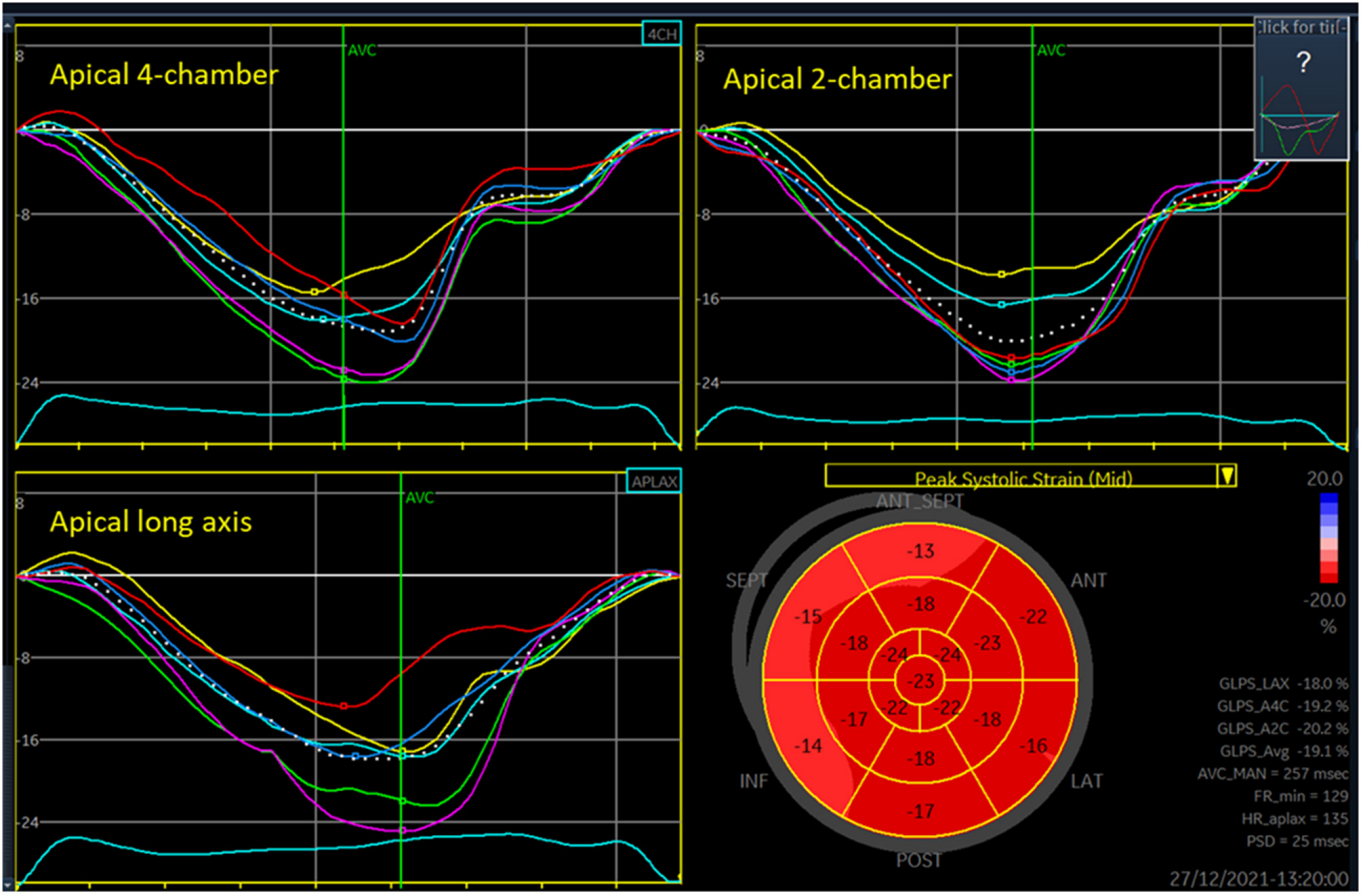
Three-plane longitudinal strain analysis of the left ventricle. Each of the panels for the three views shows seven curves, one colored curve from each of the six segments analyzed in each view and a white dotted curve for the entire region of interest. The low right panel shows values for each segment in a 17-segment plot, with basal segments at the outer edge and apical segments at the center of the figure (“bullseye plot”). The peak of each of the dotted curves is denoted GLPS (global longitudinal peak strain) for LAX (long axis), A4C (apical four-chamber), and A2C (apical two-chamber) views. The GLPS_Avg is the average of these three peaks, often referred as the global longitudinal strain. FR_min is the lowest grayscale frame rate (Hz) among the three views analyzed. HR_aplax is the heart rate in the apical long axis recording (beats per minute). PSD is the standard deviation of the time from onset of systole to segmental peak strain for each of the segments, often denoted as mechanical dispersion.

Speckle tracking echocardiography (2D strain) can assess myocardial deformation by the indexes strain and strain rate. Strain is change in the length of myocardial wall segments, relative to the length at end-diastole. Strain rate is (change in) strain per unit of time. Strain is widely used in neonatal research and is on the rise in clinical care as well. Although peaks of strain rate are short lasting and under-sampling due to low time resolution, hence, might be a problem, neonatal research papers frequently report strain rate values. Apical images enable longitudinal deformation analyses (Figure 1). Parasternal short-axis views enable circumferential and radial deformation analyses and assessment of rotational mechanics by indexes such as rotation, twist, and torsion, and their corresponding rotation rates and twist rates (4).

#### Clinical Aspects

Cavity indexes are load dependent. Shortening fraction is less sensitive than other indexes of heart function (27). Despite these weaknesses, shortening fraction is still the most commonly used index of left ventricular (systolic) function in newborns. Normal values are relatively independent of weight and gestational age, within the range of 28–45% (28). The Simpson biplane ejection fraction has replaced shortening fraction in adults, and the use of this index increases in newborns. Normal values are relatively independent of weight and gestational age and considered normal if higher than 50–55%. For the right ventricle, there is no single well-established cavity index for heart function.

MPI increases with high afterload and low preload, and, importantly, experimental studies have rarely shown that MPI changes with altered contractility. Typical MPI in preterm infants are 0.35–0.45 and in term infants, 0.3–0.4.

TAPSE and MAPSE are load- and angle-dependent ventricular function indexes and are prone to disturbances from tethering effects. Large values correspond to better function and increase with heart size. The most relevant tissue Doppler velocities of the atrioventricular plane are the peak velocity during systole (s′, “s prime”) and the e′ and a′ peak velocities at the early and late peaks in the filling phase. The velocity at peak systole (s′) is a heart function index less affected by load than most other indexes. e′ and a′ peaks assess diastolic function. In general, higher velocities are marker of better function. Importantly, normal values increase with increased heart size. Tissue Doppler velocity measurements are angle dependent and prone to disturbances from tethering effects but less load dependent than cavity indexes, TAPSE and MAPSE. Many factors influence the diastolic velocities.

Longitudinal strain has a (negative) peak value around end of systole, whereas longitudinal strain rate has three peaks: one negative peak during systole (analogous to the s′ velocity peak of the atrioventricular plane by tissue Doppler) and two peaks in diastole (analogous to e′ and a′ velocity peaks of the atrioventricular plane). In general, higher absolute values are markers of better function. Left-sided absolute strain higher than 18–20% and right-sided higher than 20–22% suggest good heart function. Loading conditions have similarly high impact on longitudinal strain as on TAPSE and MAPSE and, similarly, little impact on longitudinal peak systolic strain rate as on s′ by tissue Doppler. Importantly, heart size, angle errors, and tethering effects pose less impact on strain and strain rate than on TAPSE, MAPSE, and tissue Doppler velocities. Myocardial work is an index developed in adult cardiology. It uses longitudinal strain and estimates of afterload from the left ventricle and is hence an index of left ventricle strain adjusted for afterload. Myocardial work has been reported in neonates (29). The method probably needs more validation in small hearts, in particular related to the within-software estimates of pressure–volume curves and wall tension.

Antegrade blood flow over the atrioventricular valves has two peaks, namely, the E (early) and the A (atrial) peak. A higher A than E peak may indicate reduced (diastolic) function. The index is, however, less feasible in neonates because the E and A peaks often merge at the high heart rates in neonates and because neonatal hearts where the heart function is considered normal often exhibit peak A higher than peak E.

The fraction of blood flow E/tissue Doppler e′ of the right and the left hinges of the atrioventricular plane (left and right E/e′, “E over e prime”) are on the rise in children and newborns as indexes of diastolic function. E/e′ has been studied more extensively in children than in neonates. A low E/e′ indicates low/normal filling pressure, preload, and diastolic function, whereas high E/e′ may indicate a high filling pressure, high preload, or reduced diastolic function (30). E/e′ are prone to the above-mentioned errors of blood velocity measurements and tissue Doppler velocity measurements.

Heart function is an interplay between preload, contractility, and afterload (31, 32). The impact from pre- and afterload on measurements varies between indexes. Generally, load has less impact on indexes of myocardial wall motion (tissue velocities and deformation) than on indexes of blood flow and cavity changes. Similarly, load has less impact on indexes based on dynamic changes occurring during systole (systolic peak velocities and strain rate) than on indexes based on changes in volumes and changes between end-diastole and end-systole. Considering E/e′ as markers of preload, tricuspid valve regurgitation velocity and PAAT/RVET as markers of right-ventricle afterload, and systolic systemic blood pressure as markers of left-ventricle afterload may allow a physiological-based evaluation of ventricle function based on the various ventricular-based indexes (Table 1). Importantly, several pathological states often co-exist. One principal pathological state can lead to secondary pathological states, and their net effect on indexes may vary. As an example, the primary pathological state in a neonate with “persistent pulmonary hypertension of the newborn” is high right heart afterload due to high pulmonary vascular resistance. Secondary pathological states may include low right ventricular systolic function (due to impaired coronary perfusion). The high right ventricular afterload tends to decrease the PAAT/RVET, whereas the reduced right ventricular contractility (due to impaired coronary perfusion) tends to increase the index. The net effect can, hence, be a low or normal PAAT/RVET.

**Table 1:** Expected changes in neonates with circulatory compromise.

Principal pathological state^*^		**Low preload**	**Low diastolic function**	**Low systolic function (contractility)**	**High afterload**
**Index**					
E/e′		Low	High	Normal/high	Normal/high
Peak systolic strain rate (absolute values)		Normal	Normal	Low	Normal
s′ of the atrioventricular plane		Normal	Normal	Low	Normal
Peak strain (absolute values)		Low/normal	Low/normal	Low	Low
Right heart	TAPSE, FAC	Low/normal	Low/normal	Low	Low
Left heart	MAPSE, SF, EF	Low/normal	Low/normal	Low	Low
Right heart	PAAT/RVET	Normal	Normal	Normal	Low
	Tricuspid valve regurgitation velocity	Normal	Normal	Normal	High
Left heart	Systolic blood pressure	Low/normal	Low/normal	Low/normal	High

* *Several pathological states often appear simultaneously, and one principal pathological state can lead to secondary pathological states. Their net effect on indexes may vary; see text for details*.

*E, early diastolic peak blood flow velocity over the left and right atrioventricular valves; e′, early diastolic peak velocity for the left and right hinge of the atrioventricular valves by tissue Doppler; EF, ejection fraction; FAC, fractional area change; MAPSE, mitral annular plane systolic excursion; PAAT, pulmonary arterial acceleration time; RVET, right ventricular ejection time; s′, systolic peak tissue velocity for the left and right hinge of the atrioventricular valves by tissue Doppler; SF, shortening fraction; TAPSE, tricuspid annular plane systolic excursion*.

### Pericardial Fluid

Pericardial effusions may increase the extracardiac pressure within the pericardial cavity and hence influence filling of the heart. Large amounts of extracardiac fluid impairs cardiac filling. In patients with rapidly developing effusions, the extracardiac pressure might be higher than for effusions evolving slowly. Rapid evolving effusions, hence, may have stronger impact on cardiac filling. Intra-thoracic pressure varies by respiration, and its effects on ventricular filling becomes more evident if pericardial effusions are present. Changes with respiration of more than 30% in mitral blood flow E-velocity and in aortic stroke volumes and of more than 50% in tricuspid valve blood flow E-velocity indicate that the pressure in the pericardial cavity affects the filling of the heart enough to consider a pericardial drainage procedure.

### Catheter Position

It is possible to use NPE to assess the position of catheters in vessels. Imaging the catheter tip from multiple views and use of injected saline boluses may facilitate reliable identification of the catheter position.

### Persisting Ductus Arteriosus and Foramen Ovale

It is challenging to evaluate systemic blood flow in the presence of fetal shunts and the impact from fetal shunts on the systemic blood flow. Both the size and direction of blood flow in the shunts are important. Reversed diastolic blood flow in cerebral arteries and abdominal aorta, and antegrade diastolic blood flow in pulmonary arteries, are associated with large shunt volume in ductus arteriosus. Following ligation of the ductus arteriosus, systemic blood flow may drop due to reduced left-ventricle preload. This might lead to systemic hypo-perfusion, and many recommend intervention if systemic blood flow becomes lower than 200 ml/kg/min.

## Summary

If the information obtainable by NPE had been accessible easily (e.g., *via* a sensor put on the neonate similarly to a pulse oximeter), NPE would have been more widely used. Acquiring skills for NPE takes time and resources. Future developments in NPE probably include a stronger focus on education, training, and certification. It is likely that echocardiographic methods will evolve further, probably involving establishing new indexes and methods and implementing artificial intelligence in the analyses procedure to improve accuracy and workflow. It is important to acknowledge that NPE is not a therapeutic intervention; it is a diagnostic tool providing additional information. As it would be difficult to show in a strict scientific manner that use of pulse oximetry (or stethoscope) improves outcomes in the neonatal intensive care unit (NICU), it is challenging to show that NPE improves outcome. However, neonatologists prefer having information obtained by NPE available and NPE changes management in the NICU (33, 34). NPE has hence been one of the important developments in neonatal intensive care over the last years and will probably become an even more important tool in the years to come.

## Author Contributions

EN concepted the manuscript, wrote the text, and created the figures and the table.

## Conflict of Interest

The author declares that the research was conducted in the absence of any commercial or financial relationships that could be construed as a potential conflict of interest.

## Publisher's Note

All claims expressed in this article are solely those of the authors and do not necessarily represent those of their affiliated organizations, or those of the publisher, the editors and the reviewers. Any product that may be evaluated in this article, or claim that may be made by its manufacturer, is not guaranteed or endorsed by the publisher.
