# Supplementary material for: Neonatologist Performed Echocardiography for Evaluating the Newborn Infant
Source: Front Pediatr. 2022 Mar 24;10:853205. doi: 10.3389/fped.2022.853205 (PMC8987714; doi:10.3389/fped.2022.853205)

Supplemental figure s1

A: Assessment of Shortening Fraction, calculated as the relative change in diameter of the left ventricle from end of diastole (yellow) to end of systole (orange). The blue bar shows the line of assessment in the parasternal short-axis image.

B: Assessment of areas for left ventricular biplane Ejection Fraction by the Simpson method. Apical 4-chamber view (upper panels) and apical 2-chamber view (lower panels). Areas at end of diastole (yellow) and end of systole (orange). Biplane calculation by the Simpson method involves calculating the ventricle volumes from area changes between end of diastole and end of systole.

C: Assessment of right ventricular Fractional Area Change. Apical 4-chamber view shown, focused on the right ventricular cavity (probe rotated slightly counter-clockwise from standard 4-chamber view and tilted towards the right ventricle cavity). Fractional Area Change is the relative change in area from end of diastole (yellow) to end of systole (orange).

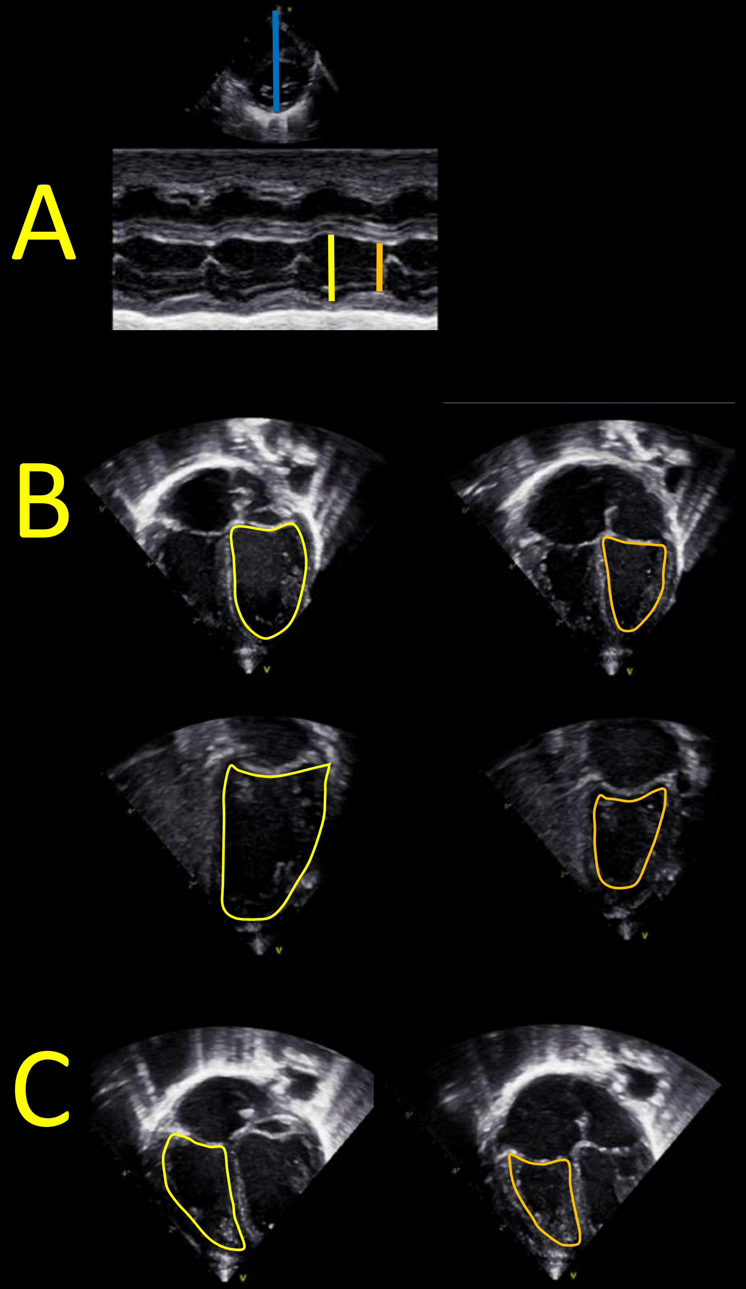

Supplemental figure s2

A: Assessment of vena cava superior flow. The velocity-time curve (first panel) has one peak at atrial systole (a, can be negative or positive), and two positive peaks, one in systole (s) and one in diastole (d). Diameter of the vessel can be assessed by m-mode (yellow bar in the second panel) or parasternal longitudinal view (yellow bar in the third panel). Cross-sectional area of the vessel can be assessed directly on a grey-scale parasternal short-axis image (last panel). To improve measurement accuracy, velocity-time curves should be assessed over several heart beats, and cross-sectional areas should be calculated from several systolic and diastolic measurements. Significant reversed a-wave velocities should be taken into account for the flow assessment.
VCS: Vena Cava Superior.

B: Assessment of aortic valve flow. The velocity-time curve (first panel) has one peak during systole. Diameter of the aortic orifice is assessed from the parasternal long-axis view (yellow bar in the second panel).

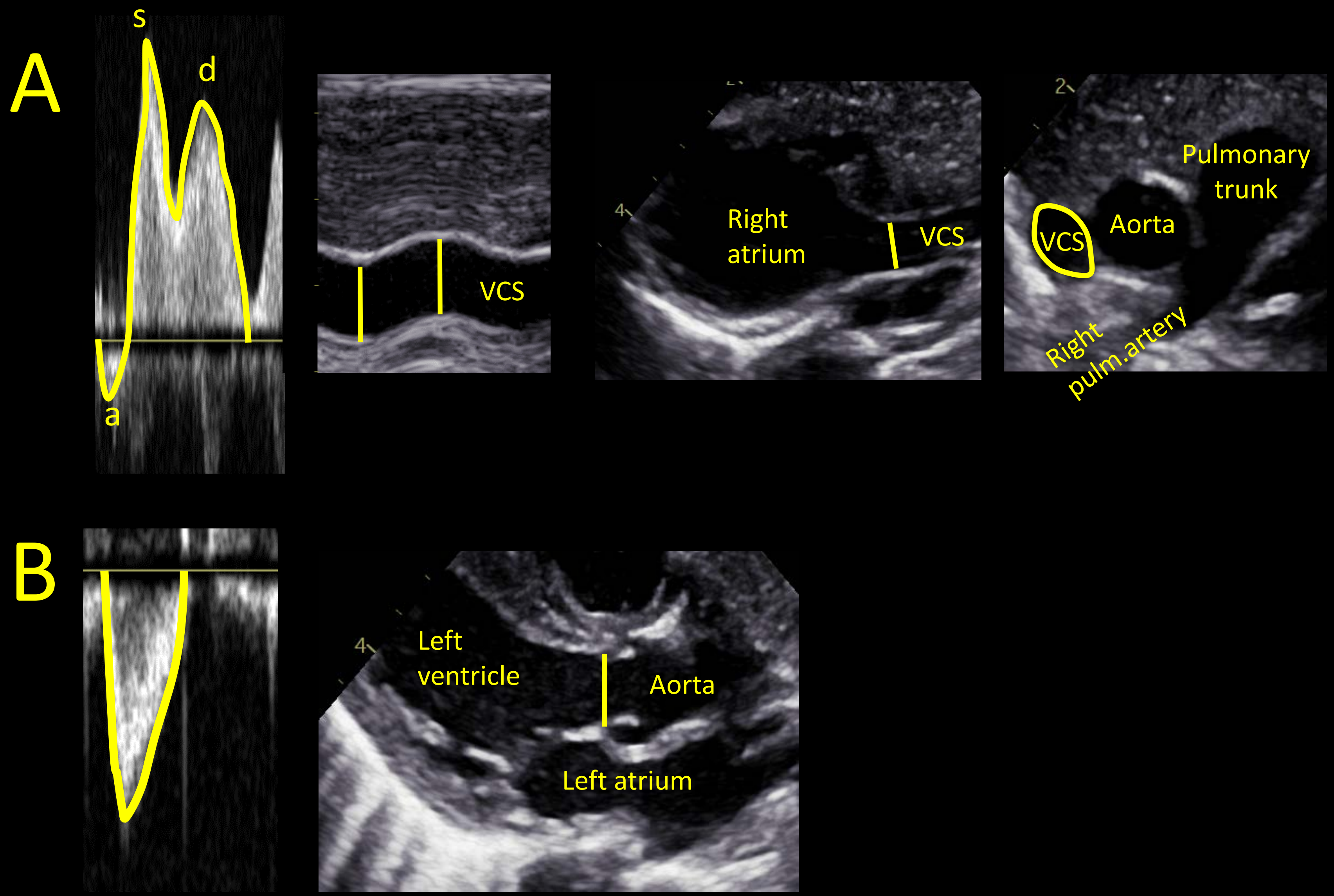

Supplemental figure s3

The ratio between Pulmonary Artery Acceleration Time (PAAT) and Right Ventricle Ejection Time (RVET) in a neonate with elevated (panel A) and normal (Panel B) pulmonary vascular resistance and pressure. Values larger than 0.31 are considered normal and below 0.23 a marker of high pulmonary vascular resistance. In panel A the PAAT is 48 milliseconds and RVET 244 milliseconds, and the PAAT/RVET is low (0.20). In panel B the PAAT is 74 milliseconds and the RVET 208 milliseconds, and the PAAT/RVET is normal (0.36).

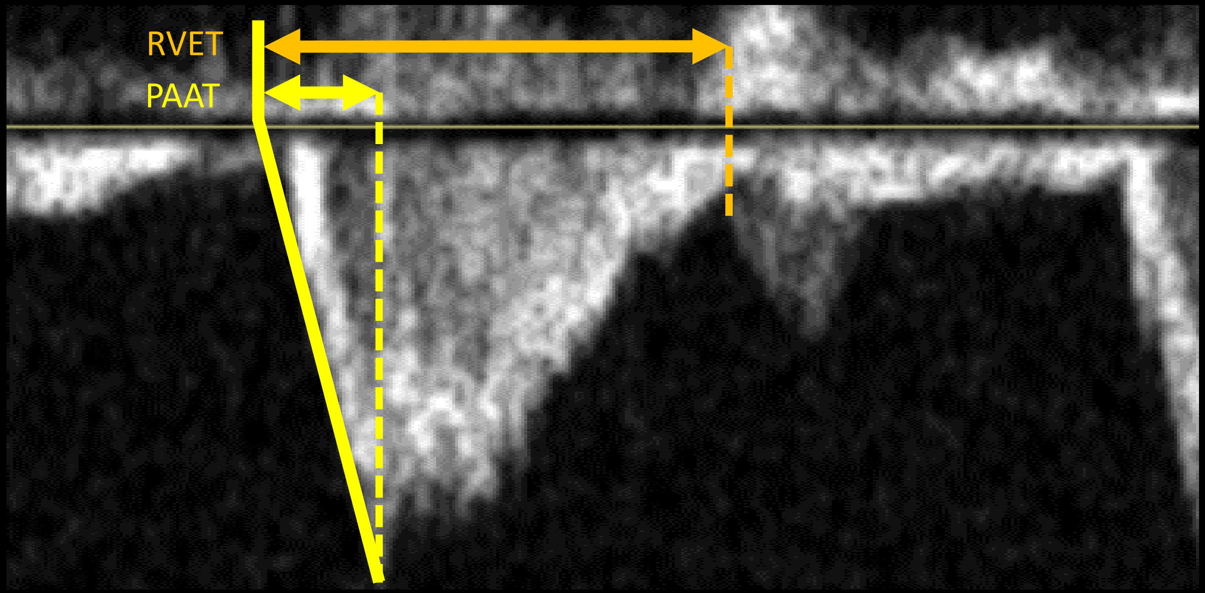

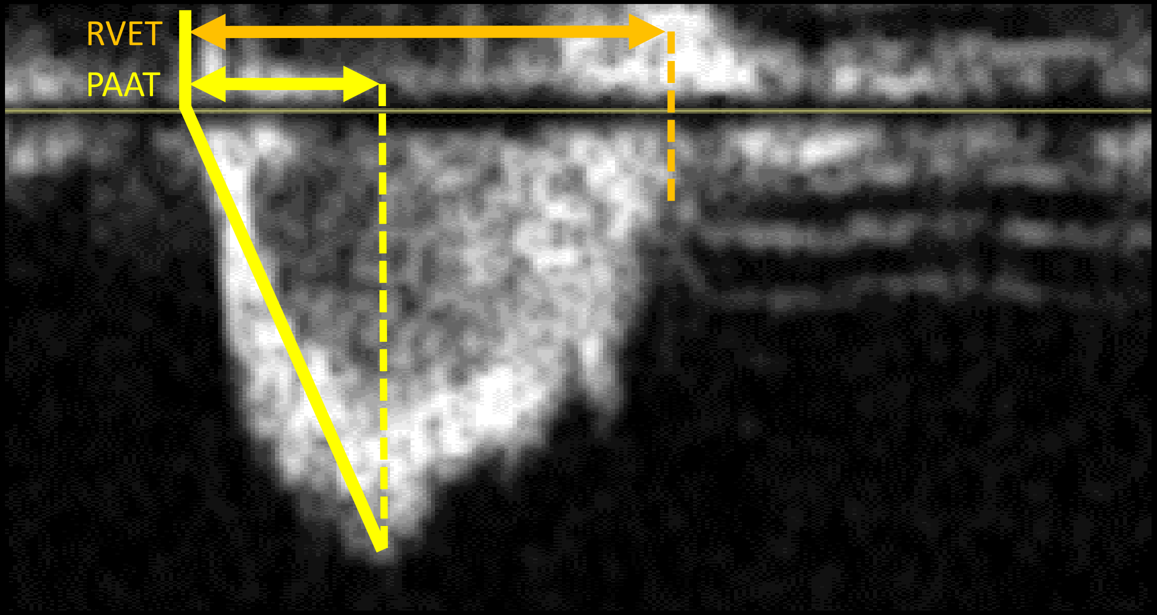

Supplemental figure s4

Pulsed-wave tissue Doppler curve from the atrioventricular plane at the lateral hinge of the tricuspidal valve. Yellow line at onset of ventricular systole. Orange bars at onset and end of the ejection phase, and onset of the filling phase. «x» denote peak velocities during systole (s'), early diastole (e') and late diastole (a').
IVC: Isovolumic contraction phase.
IVR: Isovolumic relaxation phase.

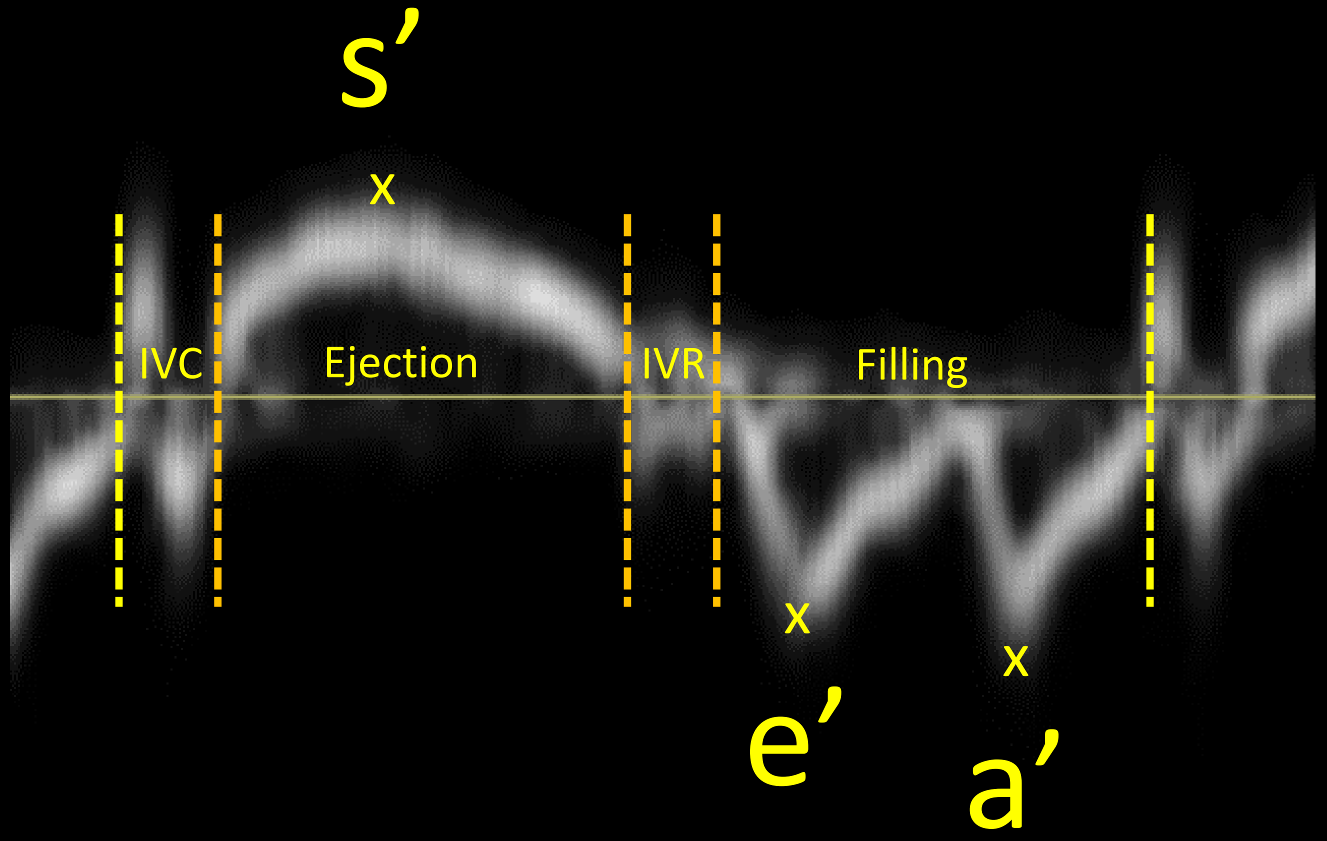

Supplemental figure s5

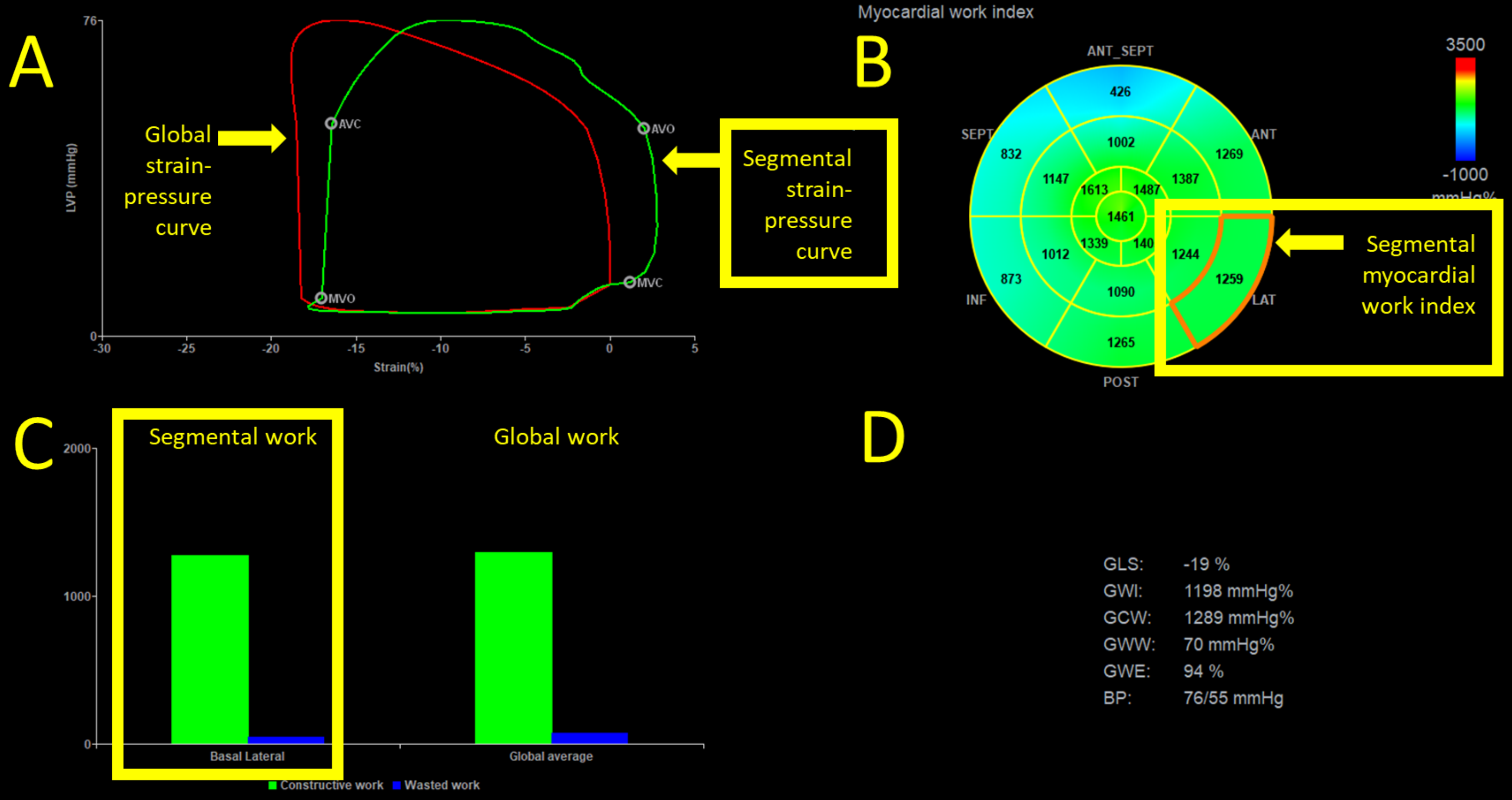

Assessment of myocardial work index of the left ventricle. Panel A shows the global strain –pressure curve (red) and a segmental strain-pressure curve (green). The analysis software creates these curves by integrating systolic blood pressure and timing of opening and closure of the aortic and mitral valves with the global and segmental strain curves. Panel B shows the myocardial work index in each left ventricular segment in a bullseye plot. The segment marked by red border is the segment show as segmental strain-pressure loop in panel A and as segmental work in panel C. Panel C shows the constructive and wasted (non-constructive) work in the segment (left bars) and the entire left ventricle (right bars). Panel D: Global myocardial work indexes for the left ventricle.

Supplemental figure s6

Blood speckle imaging in an image of the left ventricle from the apical long axis view. The lines denote direction of blood movement during the cardiac cycle. Upper panel shows laminar flow into the ventricle cavity during the early filling phase of the diastole (E-wave filling). Mid panel shows laminar flow during the atrial systole (A-wave filling), with a circular movement of blood in the outflow tract of the ventricle. Lower panel shows laminar flow in the outflow tract into the aorta during systole.
LA: Left atrium
LV: Left ventricle
Ao: Aorta

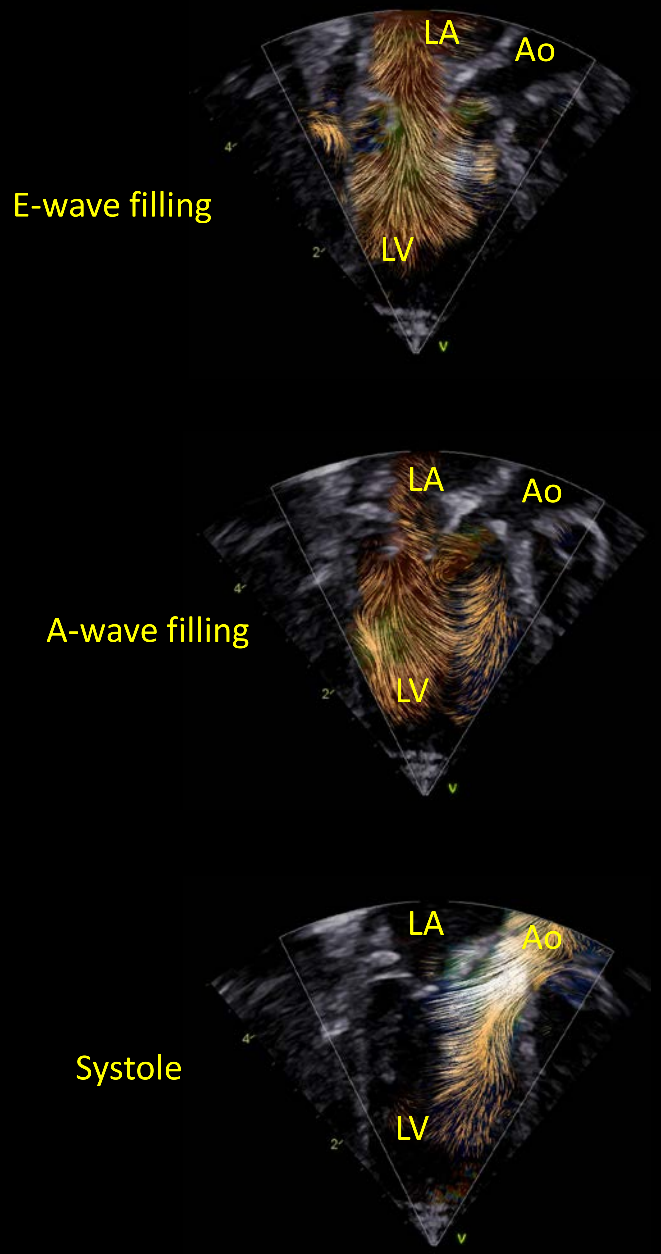

Supplemental figure s7

Example of an experimental technique, three-dimensional assessment of right ventricular cavity volumes by echocardiography. It uses real-time one-cycle recordings. By post-recording processing (left panels) the software creates a three-dimensional model of the right ventricular cavity (upper right panel) and estimates ventricular volumes during the cardiac cycle (lower right panel). Each yellow dot in the low right panel denote one frame of the recording, showing the relatively low time resolution. In this example there are 11 frames (timpepoints) during the heart cycle where the software calculates the volume of the cavity.

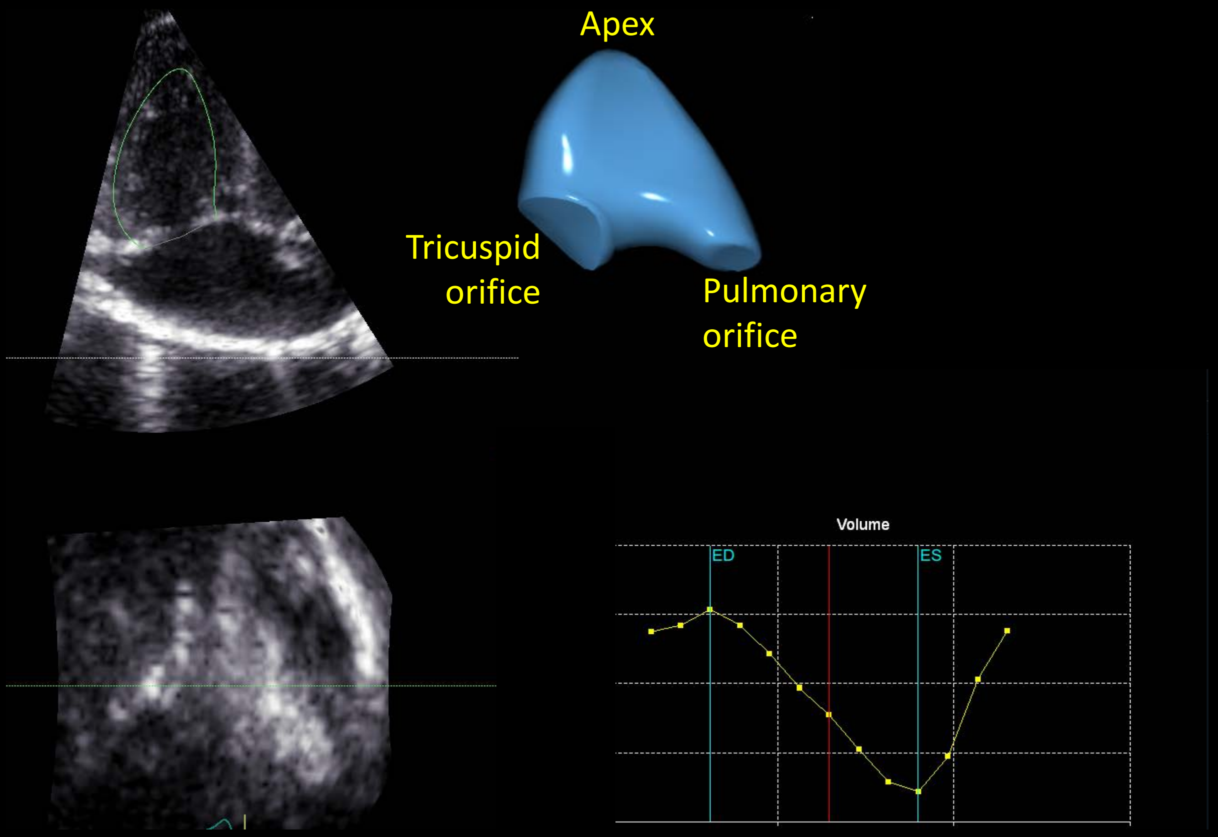

Supplement: Supplementary file 1 [file Presentation_1.pdf]
